# Peripheral Maintenance of the Axis SIRT1-SIRT3 at Youth Level May Contribute to Brain Resilience in Middle-Aged Amateur Rugby Players

**DOI:** 10.3389/fnagi.2019.00352

**Published:** 2019-12-17

**Authors:** Rubén Corpas, Elisabeth Solana, Adrian De la Rosa, Sara Sarroca, Christian Griñán-Ferré, Mireia Oriol, Emili Corbella, Eduard Rodríguez-Farré, Jose Vina, Mercè Pallàs, David Bartrés-Faz, Mari Carmen Gomez-Cabrera, Coral Sanfeliu

**Affiliations:** ^1^Institut d’Investigacions Biomèdiques de Barcelona (IIBB), Consejo Superior de Investigaciones Científicas (CSIC), Institut d’Investigacions Biomèdiques August Pi i Sunyer (IDIBAPS), Biomedical Research Centre Network for Epidemiology and Public Health (CIBERESP), Barcelona, Spain; ^2^Departament de Medicina, Facultat de Medicina i Ciències de la Salut, Institut de Neurociències, Universitat de Barcelona, IDIBAPS, Barcelona, Spain; ^3^Freshage Research Group, Department of Physiology, Faculty of Medicine, University of Valencia, Centro de Investigación Biomédica en Red Fragilidad y Envejecimiento Saludable (CIBERFES), Fundación Investigación Hospital Clínico Universitario/INCLIVA, Valencia, Spain; ^4^Unitat de Farmacologia i Farmacognòsia, Facultat de Farmàcia i Ciències de l’Alimentació, Institut de Neurociències, Universitat de Barcelona, Barcelona, Spain; ^5^Clinic Institute of Nephrology and Urology (ICNU), Hospital Clínic, Barcelona, Spain; ^6^Unitat de Risc Vascular Medicina Interna, Hospital Universitari de Bellvitge, IDIBELL, CIBER Fisiopatología Obesidad y Nutrición (CIBEROBN), Barcelona, Spain

**Keywords:** physical exercise, middle-aged and young men, whole-blood gene expression, SIRT1, SIRT3, brain resilience

## Abstract

Physical exercise performed regularly is known to improve health and to reduce the risk of age-related diseases. Furthermore, there is some evidence of cognitive improvement in physically active middle-aged and older adults. We hypothesized that long-term physically active middle-aged men may have developed brain resilience that can be detected with the analysis of peripheral blood markers. We aimed to analyze the activation of pathways potentially modulated by physical activity in a cohort of healthy amateur rugby players (*n* = 24) and control subjects with low physical activity (*n* = 25) aged 45–65 years. We had previously reported neuropsychological improvement in immediate memory responses in the player group compared to the controls. Here, we tested the expression of selected genes of longevity, inflammation, redox homeostasis, and trophic signaling in whole blood mRNA. Analyses were also performed on blood samples of young (aged 15–25 years) control subjects with low physical activity (*n* = 21). Physical activity and other lifestyle factors were thoroughly recorded with standardized questionnaires. Interestingly, middle-aged control subjects showed lower levels of expression of SIRT1, SIRT3, CAT, and SOD1 than the young controls, although rugby players maintained the expression levels of these genes at a young-like level. Middle-aged players showed lower levels of IL1B than the non-physically active groups. However, there was a tendency towards a decrease in trophic and transduction factors in middle-aged groups as compared to the young controls. A statistical study of Spearman’s correlations supported a positive effect of sporting activity on memory and executive functions, and on peripheral gene expression of SIRT1, SIRT3 and downstream genes, in the middle-aged rugby players. Our results indicate that the SIRT1-SIRT3 axis, and associated neuroprotective signaling, may contribute to the anti-aging resilience of the brain mediated by physical exercise.

## Introduction

Health-promoting lifestyles have been proposed as a means of improving wellbeing, counteracting frailty and of delaying organismal aging and death. The combination of several healthy behaviors, that include a diet rich in fruits and vegetables, moderate alcohol consumption, non-smoking and regular physical activity, was reported to add a level of rejuvenation equivalent to 14 years in chronological age (Khaw et al., 2008). Similarly, lifestyles which include physical activity have proven to delay age-related cognitive impairment and decrease the risk of Alzheimer’s disease (AD) and vascular dementia (Gallaway et al., 2017; Lin et al., 2019). Age-related processes of cell and tissue deterioration (López-Otín et al., 2013) are common in brain and peripheral organs. Furthermore, preservation of cell-survival mechanisms may induce milder AD phenotype (Sarroca et al., 2016). Conversely, frailty increases the risk of AD severity (Wallace et al., 2019). Therefore, we may speculate that healthy behaviors acquired early and maintained throughout a lifetime can build brain and body resilience against age-related ailments. Resilience, reserve, and resistance are concepts used in brain aging and AD to identify preventive interventions (Montine et al., 2019). The concepts of cognitive reserve and brain resilience similarly refer to the ability to maintain functional networks despite higher than expected brain pathological changes, whereas resistance refers to the ability of having lower than expected pathological changes (Negash et al., 2013; Arenaza-Urquijo and Vemuri, 2018). Furthermore, we hypothesized that resilience might be detected at early healthy aging when minor deterioration of the body and mind starts to show in subjects in their 50s.

In a recent study, we demonstrated that a cohort of middle-aged, amateur male rugby players showed physiological adaption to long-term physical activity. Namely, they showed lower resting levels in circulating brain-derived neurotrophic factor (BDNF) than aged-matched subjects with lower physical activity. Similar results were obtained from the simultaneous analyses of young men who regularly practiced sports vs. age-matched sedentary men (De la Rosa et al., 2019). Interestingly, the veteran rugby players scored slightly but significantly higher statistically than the age-matched controls in neuropsychological tests of declarative verbal memory (De la Rosa et al., 2019). Therefore, this middle-aged collective could help to discern differential markers of early cognitive aging linked to physical activity-induced resilience. Exercise training activates transcriptional regulators and enzymes to reprogram many pathways in multiple adaptive organismal responses. We aimed to analyze peripheral blood samples for changes in gene expression in the main pathways related to physical activity as described below:

(a) Physical exercise activates cell mechanisms, regulating energy expenditure that increases nicotinamide adenine dinucleotide (NAD^+^) levels in response to the higher intracellular AMP/ATP ratio (Cantó et al., 2009). Increases in NAD^+^ will induce the activation of the NAD^+^-dependent histone deacetylase sirtuin family of proteins that improves healthspan (Haigis and Sinclair, 2010). Sirtuins (SIRT1-SIRT7) deacetylate, and therefore activate, many enzymes and transcription factors crucial for maintaining cell physiology, stress response, and survival signaling. The sirtuins examined in this study, SIRT1, SIRT2, SIRT3, and SIRT6, are potential targets in the fight against brain aging and neurodegeneration (Satoh et al., 2017). SIRT1 and SIRT6 are preferentially located in the nucleus, SIRT3 in mitochondria and SIRT2 in the cytosol; all of them are expressed in a variety of body tissues and organs including the brain, blood cells and skeletal muscle (Grabowska et al., 2017).
(b) Increased oxidative damage is one hallmark of tissue and organ aging. It has long been known that antioxidant enzymes are up-regulated by physical exercise as a hormetic response to bursts of oxidative stress caused by increased metabolic activity (Radak et al., 2008). Some reports show discrepancies as to whether such enzymes vary with aging or that such aging is tissue-dependent (Zhang et al., 2015). However, some decreases might contribute to age-associated oxidative stress. Habitually doing physical exercise at an old age may prevent the establishment of age-related oxidative stress (Pierce et al., 2011) and reduce immunosenescence features (Duggal et al., 2018). We analyzed the genes CAT, SOD1, SOD2, GPX1 and GPX4, codifying for catalase, Cu/Zn superoxide dismutase, Mn superoxide dismutase, and glutathione peroxidase isoforms 1 and 4, respectively. The gene NFE2L2 codes for the transduction factor Nuclear factor (erythroid-derived 2)-like 2 (i.e., Nrf2), that regulates the expression of detoxification and antioxidant genes. Sirtuins may activate the expression of antioxidant genes through the deacetylation of Nrf2 (Singh et al., 2018). Additionally, SIRT1 may promote the expression of SOD2, CAT, and other antioxidant genes through the deacetylation of FOXO3a and PGC-1α transcription factors (Olmos et al., 2013). Furthermore, SIRT3 post-translationally activates SOD2 and other mitochondrial enzymes in response to the presence of reactive oxygen species (Chen et al., 2011).
(c) There is chronic low-grade systemic inflammation in aging that contributes to the risk of age-related illnesses and memory loss (Bradburn et al., 2018; Rea et al., 2018). Regular exercise downregulates several pro-inflammatory pathways in the elderly (Woods et al., 2012). We analyzed gene expression of the pro-inflammatory cytokines interleukin 1β (IL1B) and interleukin 6 (IL6) and the anti-inflammatory cytokine IL10, all widely characterized in health and disease. SIRT1 may directly inactivate NF-kB; SIRT6 may do it indirectly (i.e., through intermediate processes; Yeung et al., 2004; Kawahara et al., 2009). Therefore, sirtuins may contribute to decreasing inflammation. Furthermore, changes in redox homeostasis may regulate inflammatory genes (Lavrovsky et al., 2000).
(d) Lastly, physical activity promotes trophic factor signaling (Cobianchi et al., 2017). Myokines such cathepsin B (CTSB; Moon et al., 2016), vascular factors such as vascular endothelium growth factor A (VEGFA) and other peripheral factors induced by exercise may have a positive impact on brain tissue. One of the best characterized is the BDNF also synthesized in the brain (Marosi and Mattson, 2014). The cAMP response element-binding protein 1 (CREB1) is a major mediator of neurotrophin responses.

All of these genes are expressed in blood cells and we analyzed their levels in middle-aged amateur rugby players and age-matched controls, adding a group of young subjects as age control. Subsequently, we searched for correlations in gene expression with the previously reported changes in declarative verbal memory (De la Rosa et al., 2019) and with other neuropsychological data that did not showed significant changes between both middle-aged groups. We also searched for correlations with behavior potentially related to health and resilience. For this purpose, we examined physical activity and other lifestyles and health behaviors through standardized questionnaires. We suggest that these analyses may help to discern genes or gene pathways involved in the promotion of brain resilience.

## Materials and Methods

### Study Design and Participants

Middle-aged amateur rugby players participating in veterans’ competitions (males, *N* = 24), age-matched controls with low physical activity (males, *N* = 25) and young controls with low physical activity (males, *N* = 21) were enrolled in a study approved by the Ethics Committee of the Hospital Clínic de Barcelona, Spain (Reg. HCB/2014/0759). Written informed consent was obtained from all participants in the study in accordance with the Declaration of Helsinki. The rugby players were long-term practitioners of the sport, engaged in weekly training and game playing for an average period of 35 years (range: 7–59 years). Control subjects did either no sports or leisure physical activity at all, or they performed such activities for less than 150 min per week, as recorded in the STEPS questionnaire (see below). Reasons for exclusion from the study included: consumption of neuroactive drugs, medical histories of brain disease or other serious health conditions or moderate or severe traumatic brain injury. Mild traumatic brain injuries, namely concussions, were recorded. Subjects were recruited during 2014 and 2015. Neuropsychological testing and blood sampling were performed in 2015. A previous study with this cohort is reported in De la Rosa et al. (2019).

All subjects were interviewed by a neuropsychologist trained in clinical studies and were also asked to fill out self-administered questionnaires. Middle-aged subjects were tested with a battery of neuropsychological tests. All tests and questionnaires were validated and recognized in the field. Blood samples were obtained by a trained nurse, processed and stored at −80°C until use. The support of the Mario Sàlvia Foundation of the Institut d’Estudis Catalans allowed the Cerebral Aging and Lifestyles (*Envelliment Cerebral i Estils de Vida*, ECEV) sample collection to be established in the National Registry of Biobanks, from the Instituto de Salud Carlos III, Spain.

### Physical Activity and Lifestyle Questionnaires

All subjects of the study were asked to fill out the following questionnaires:

(a) WHO STEPwise approach to chronic disease risk factor surveillance (STEPS) questionnaire, core information. Information to be filled out includes the health variables of hypertension, hyperglycemia, body height, and weight, smoking habits, diet habits (including alcohol consumption), and physical activity habits (WHO STEPS Surveillance Manual, 2008). Degrees of physical activity was given as hours spent doing the activity in a representative week.
(b) International Physical Activity Questionnaire (IPAQ), long version. Information on the health-related physical activity refers to the last 7 days (Craig et al., 2003). Results were calculated as the weekly metabolic equivalent of a task in minutes (MET-min) for the different categories of physical activity.
(c) Minnesota Leisure-Time Physical Activity Questionnaire (MLTPAQ) quantifies the metabolic rate of different leisure physical activities performed during the last 12 months (Folsom et al., 1986). Values were calculated as the yearly total MET-h.

### Psychological Questionnaires

All subjects of the study were asked to fill out the following scales and questionnaires:

(a) Hamilton Rating Scale for Anxiety (Hamilton-A). Rates 14 groups of symptoms of anxiety, on a scale of 0–5 points. Composite scores of 0–17 indicate absence of or mild anxiety; 18–24 indicate mild to moderate anxiety; and 24–30 indicate moderate to severe anxiety (Hamilton, 1959).
(b) Hamilton Rating Scale for Depression (Hamilton-D). Rates 17 symptoms of depression on a scale of 0 and 3 points. Scores of 0–7 indicate the absence of depression and 20 or higher indicate at least moderate depression (Hamilton, 1960).
(c) Pittsburgh Sleep Quality Index (PSQI). Rates seven components of sleep quality on a scale of 0–3. The overall score ranges from 0 to 21, with low scores of 0–5 indicating good sleep quality (Buysse et al., 1989).
(d) Memory Functioning Questionnaire (MFQ). A 7-item scale, with each item rated from 1 to 7, including different aspects of self-appraisal of memory, forgetfulness and mnemonics usage. Higher composite scores indicate better self-appraised memory functioning (Gilewski et al., 1990).
(e) Cognitive Reserve Variables Questionnaire. Gives a composite score for the presence of lifestyles considered to increase cognitive reserve including education, occupational work and leisure activities of intellectual, social and physical activity type (Solé-Padullés et al., 2009). In the validation of this cognitive reserve score, the authors reported that cognitively-healthy adults older than 65 years scored 7–11, whereas cognitively-impaired subjects scored 3–7 (Solé-Padullés et al., 2009).

### Blood Collection and Extraction of DNA and RNA Samples

Peripheral blood samples from all the subjects were obtained from the antecubital vein following overnight fasting. Blood was collected in different tubes in accordance with the required testing methods. BD Vacutainer™ tubes (BD Diagnostics, Frankin Lanes, NJ, USA) containing anticoagulant EDTA were used for DNA analysis. Once the blood samples were collected, the tubes were gently inverted 8–10 times and then frozen at −80°C until DNA extraction. The DNA extraction was performed using the Wizard Genomic DNA Purification Kit (Promega, Fitchburg, WI, USA) in accordance with the manufacturer’s instructions. Tempus™ Blood RNA tubes (Thermo Fisher Scientific, Waltham, MA, USA) were used for the stabilization of total RNA for gene expression analysis. Once the blood samples were collected, the tubes were shaken vigorously for 10 s and frozen at −80°C until RNA extraction. Extraction was performed using the *mir*Vana™ miRNA Isolation Kit with phenol (Applied Biosystems, Foster City, CA, USA) in accordance with the manufacturer’s instructions for obtaining total RNA, including small RNA. Purity and concentration of DNA or RNA were assessed in a NanoDrop ND 1000 spectrophotometer (Thermo Fisher Scientific). Blood samples were collected the same day or within a close time frame of the neuropsychological testing for the middle-aged subjects (see below).

### Genetic Analysis

DNA samples were analyzed to determine APOE allele distribution. The APOE gene is polymorphic at two single nucleotides (rs429358 and rs7412), resulting in the alleles ε2, ε3, and ε4. APOE ε4 is considered a risk factor for various conditions associated with a cognitive loss with advancing age. It was analyzed with the quantitative Polymerase Chain Reaction (qPCR), using a combination of two TaqMan probes doubly marked with 6-carboxyfluorescein (FAM) and VIC fluorescents (Taqman SNP Genotyping Assays, Applied Biosystems), which demonstrated the different combinations of the alleles ε2, ε3 and ε4 (Zhong et al., 2016).

RNA samples were analyzed to quantify the expression of selected genes. Genes of the sirtuin family of proteins involved in longevity and neuroprotection (SIRT1, SIRT2, SIRT3 and SIRT6), antioxidant enzymes and related factors (CAT, SOD1, SOD2, GPX1, GPX4 and NFE2L2), inflammatory-related proteins (IL1B, IL6 and IL10), and trophic factors and downstream effectors (BDNF, NTRK2, CREB1, CTSB and VEGFA). For qPCR analysis, random-primed cDNA synthesis was performed using the High-Capacity cDNA Archive kit (Applied Biosystems). Gene expression was measured with specific TaqMan FAM-labeled probes (Applied Biosystems) in a CFX96 Real-Time qPCR Detection System (Bio-Rad, Hercules, CA, USA). Data were normalized to PGK1 and B2M. Results were calculated using the 2-ΔΔCt method and expressed relative to the middle-aged control group. A list of probes utilized is presented in Supplementary Table S1.

### Analysis of Superoxide Dismutase Enzymatic Activity and Interleukin 1β Protein Levels

Enzymatic activity of SOD was selected as a measure of antioxidant changes in plasma. The analysis was performed using a 19160 SOD Determination Kit (Sigma-Aldrich, Merck KGaA, Darmstadt, Germany) following the manufacturer’s instructions. The SOD activity was calculated as the inhibition rate of the reduction of a water-soluble formazan dye by superoxide anion.

The protein level of IL1B was determined as a representative pro-inflammatory cytokine in plasma using a Quantikine HS ELISA Kit (#HSLB00D; R&D Systems, Minneapolis, MN, USA).

### Neuropsychological Tests

Subjects of both middle-aged groups; controls and rugby players, were submitted to the following neuropsychological tests:

(a) Free and Cued Selective Reminding Test (FCSRT). In this test, declarative learning and memory are evaluated using a list of 16 words, which must be remembered over the course of three attempts. Both immediate and long-term memory is evaluated freely and with the help of semantic cues (Buschke, 1984). Scores are given for immediate free recall, immediate cued recall, delayed free recall and delayed cued recall. See a detailed description of the test in De la Rosa et al. (2019).
(b) Trail Making Test (TMT). In the TMT Part A, a series of numbers printed on a sheet of paper must be connected using a pen-drawn line from the smallest to the largest and at the highest possible speed. This subtest evaluates psychomotor speed and attention. In the TMT Part B, numbers and intercalated letters should be joined in numerical order and alphabetical order (Llinàs-Reglà et al., 2017). This subtest also evaluates a component of executive functions. Shorter time in seconds to complete either TMT-A or TMT-B indicates a better response.
(c) Symbol Digit Modality Test (SDMT). This test measures psychomotor speed and attention through a substitution task where geometric symbols, repeatedly listed at random, must be paired with the corresponding numbers from 1 to 9. The score is the number of correct substitutions performed in 90 s (Smith, 1982).
(d) Wechsler Adult Intelligence Scale fourth edition (WAIS-IV). The WAIS test was designed to measure intelligence and cognitive abilities in clinical practice (Wechsler, 1955). Here we used several subtests of the current version WAIS-IV. The direct digits subtest evaluates attention and working memory, the inverse digits subtest evaluates executive functions, working memory and vocabulary, as well as premorbid intellectual level.
(e) Verbal fluency. Executive functions are also assessed by verbal fluency tasks where the participants must issue as many words as possible of a given semantic category or that begin with a given letter, for 1 min (Lezak et al., 2012). The score is the number of words issued.
(f) Stroop color and word test. The automatic task of reading a list of words of color names interferes with the requested task of naming the ink color of the words; the phenomenon is known as the Stroop effect. A score of correct hits measures processing speed and the executive function of inhibition (Golden, 1978).
(g) Cambridge Neuropsychological Test Automated Battery (CANTAB). The CANTAB tests are computerized programs designed to assess several cognitive areas (Robbins et al., 1994). Here we used Spatial Working Memory (SWM), Paired Association Learning (PAL) and Rapid Visual Information Processing (RPV) tests.

### Statistics

Results are displayed as mean ± SD for normally distributed variables, median [interquartile range (IQR)] for non-normal variables and number (percentage) for qualitative variables. The Normality of distribution for quantitative variables was analyzed by the Shapiro–Wilk test. For comparisons between three groups, quantitative normal variables were analyzed by one-way ANOVA followed by Tukey’s test for multiple comparisons, and non-normal variables were analyzed by the Kruskal–Wallis test followed by Dunn’s test. Data pairs were analyzed using the two-tailed Student’s *t*-test or Mann–Whitney test as appropriate. Qualitative variables were analyzed using the Chi-square test. Bivariate correlations were analyzed with the two-tailed Spearman’s rho. Statistical analyses were performed using the GraphPad Prism v 6.01 (GraphPad, La Jolla, CA, USA) and the IBM Statistical Package for the Social Sciences (SPSS) software v 23.0 (IBM Corporation, Armonk, NY, USA).

## Results

### Characteristics of the Study Groups

Sociodemographic variables, selected habits and health parameters of the subjects enrolled in the study are shown in Table 1. Both middle-aged groups had a similar mean outcome in age and years of scholarship. Subjects were typically in their mid-fifties, with a mean age of 56.0 for the control group; including subjects from 47 to 67 years and a mean age of 54.3 years for the rugby players; including subjects from 46 to 68 years. The young control group showed a mean age of 20.9 years with subjects ranging from 17 to 24 years; some of them have not finished their years of scholarship and this parameter is not considered for this group. However, they had at the time of testing an average education level similar to that of the other groups, between high school and university level (not shown). Body mass index (BMI) and occurrences of hypertension and hyperglycemia were similar among middle-aged groups. The young subject group showed a lower BMI and lower incidences of hypertension. The frequency of APOE allele ε4 was similar among all groups. No subject presented the form ε4 for both alleles of the gene. Frequency of all three APOE alleles and the resulting genotypes (not shown) was in agreement with the general distribution in the population (Corbo and Scacchi, 1999). The rugby player group showed higher incidences of concussion than the age-matched control group. Habits of smoking and diet were similar among groups, albeit with a slightly lower fruit intake in young controls. The levels of anxiety, depression, and quality of sleep (as analyzed with the self-administered questionnaires Hamilton-A, Hamilton-D, PSQI, and MFQ, respectively) of all subjects assessed were within the normal range. No differences were detected among the study groups, but a lower subjective memory function of young controls in the MFQ. In the questionnaire of Cognitive Reserve Variables, all groups showed normal mean scores, and the significantly higher value of the rugby players is probably a reflection of their higher physical activity component.

**Table 1:** Sociodemographic, dietary habits and health status parameters.

	Middle-aged controls	Middle-aged rugby players	Young controls	*P*
	*N* = 25	*N* = 24	*N* = 21	
**Sociodemographic variables**				
Age (years)	56.0 ± 5.9	54.3 ± 6.6	20.9 ± 2.2***^,###^	<0.0001
Schooling (years)	14.4 ± 3.3	14.9 ± 3.4	ND	0.5885
**Clinical and genetic variables**				
Body mass index (Kg/m^2^)	28.4 ± 3.9	28.9 ± 2.7	24.2 ± 4.6**^,###^	0.0001
Hypertension				
Diagnosis (*N*, %)	10 (40%)	10 (41.7%)	1 (4.8%)**^,##^	0.0105
Treatment (*N*, %)	5 (20%)	5 (20.8%)	0 (0%)	0.0818
Hyperglycemia				
Diagnosis (*N*, %)	5 (20%)	3 (12.5%)	0 (0%)	0.1027
Treatment (*N*, %)	2 (8%)	1 (4.2%)	0 (0%)	0.4103
APOE ε4 (*N*, %)^1^	7 (28%)	5 (21%)	4 (19%)	0.7395
Concussion^2^ (*N*, %)	4 (16%)	12 (50%)*	ND	0.0157
**Smoking and dietary habits**				
Tobacco use (*N*, %)				
Never	9 (36%)	12 (50%)	13 (62%)	
Former	8 (32%)	8 (33.3%)	1 (4.8%)	0.0994
Current	8 (32%)	4 (16.7%)	7 (33%)
Fruit consumption (servings/week)	12 (7–21)	5 (3–14)	3 (2–14)*	0.0362
Vegetable consumption (servings/week)	6 (4–14)	5.5 (4–8.25)	8 (4–14)	0.6481
Alcohol drinking frequency^3^	3 (2–5)	3 (2–4.75)	2 (1–3)	0.1680
**Psychological variables**				
Hamilton rating scale for anxiety	8.1 ± 1.9	8.0 ± 2.0	9.4 ± 3.2	0.0926
Hamilton rating scale for depression	8.4 ± 1.4	9.1 ± 1.4	8.4 ± 2.5	0.3037
Pittsburgh sleep quality index	5.3 ± 2.6	4.7 ± 2.2	5.6 ± 1.8	0.4175
Memory functioning Q	93.8 ± 10.3	91.7 ± 13.6	84.8 ± 12.3*	0.0466
Cognitive reserve variables Q	13.8 ± 3.0	16.8 ± 3.3*	14.2 ± 2.9^##^	0.0021

Data are presented as mean ± SD, median (IQR), or number (%). Statistics: ANOVA or Kruskal–Wallis test, followed by Tukey’s test or Dunn’s test, respectively, Chi squared test, or Student’s *t*-test, where appropriated; *P < 0.05, **P < 0.01, ***P < 0.001 compared to Middle-aged control group, ^##^*P* < 0.01, ^###^*P* < 0.01 compared to Middle-aged rugby player group. ND, not determined. NOTES: ^1^subjects with one allele (no subjects with two alleles were detected; ^2^subjects with at least one episode; ^3^alcohol drinking last year: from 0, no drinking, to 5, daily intake.

### Physical Activity

The veteran rugby players performed significantly higher rates of physical activity during leisure time than the age-matched controls and young controls, as displayed in Table 2. The statistical differences were consistent in the outcome of the three questionnaires: IPAQ, STEPS, and MLTPAQ. The player group reported significantly higher values of physical activity during leisure than middle-aged and young control groups in the 1-week IPAQ questionnaire. Physical activity at work or at home and in the garden did not differ among the groups. No differences in walking or bicycling were also detected. Nevertheless, the IPAQ total MET remained higher in the player group as compared to both control groups showing median values of MET-min/week of two times the middle-aged control group and 3.2 times the young control group. Similarly, in the STEPS questionnaire, there were no differences in physical activity at work or in travel, but in leisure activities. The greatest difference between both control groups and the player group was found in the intense physical activity performed during leisure. In the 1-year records of all leisure activities in the MLTPAQ, the player group showed median values of MET-h/year of 2.2 times the middle-aged control group and 1.8 times the young control group. Rugby accounted for a third of all leisure activities of the player group in total MET.

**Table 2:** Physical activity parameters.

	Middle-aged controls	Middle-aged rugby players	Young controls	*P*
	*N* = 25	*N* = 24	*N* = 21	
**IPAQ (MET-min/week)**				
PA at work	30 (0–1,091)	0 (0–3,990)	0 (0–0)	0.2258
PA in travel, walking and bicycling	363 (181–990)	477 (132–864)	600 (330–10,539)	0.5057
PA at home or in garden	885 (0–1,541)	420 (0–1,905)	175 (80–630)	0.5738
PA during leisure	198 (0–531)	3,078 (1,440–4,386)***	0 (0–396)^###^	<0.0001
Total PA	2,298 (1,604–3,846)	4,606 (2,962–10,647)*	1,435 (990–1,886)^###^	<0.0001
**STEPS, PA (min/week)**				
Moderate PA at work	0 (0–50)	0 (0–0)	0 (0–0)	0.2944
Intense PA at work	0 (0–0)	0 (0–0)	0 (0–0)	0.3585
PA during walking and bicycling	22 (0–162)	0 (0–124)	100 (30–240)	0.1513
Moderate PA during leisure	0 (0–90)	90 (0–240)	0 (0–15)^##^	0.0089
Intense PA during leisure	0 (0–0)	240 (90–450)***	0 (0–0)^###^	<0.0001
**MLTPAQ (MET-h/year)**				
All leisure activities	1,379 (751–4,702)	3,081 (2,209–5,232)**	1,709 (935–2,583)^##^	0.0022
Rugby	-	742 (546–1181)	-	

Data are presented as median (IQR). Statistics: Kruskal–Wallis test, followed by Dunn’s test; *P < 0.05, **P < 0.01, ***P < 0.001 compared to Middle-aged control group, ^##^*P* < 0.01, ^###^*P* < 0.001 compared to Middle-aged rugby player group. Abbreviations: PA, physical activity; IPAQ, International Physical Activity Questionnaire; STEPS, WHO STEPwise Approach to Chronic Disease Risk Factor Surveillance questionnaire; MLTPAQ, Minnesota Leisure Time Physical Activity Questionnaire.

### Gene Expression in Peripheral Whole Blood

**Figure 1:**
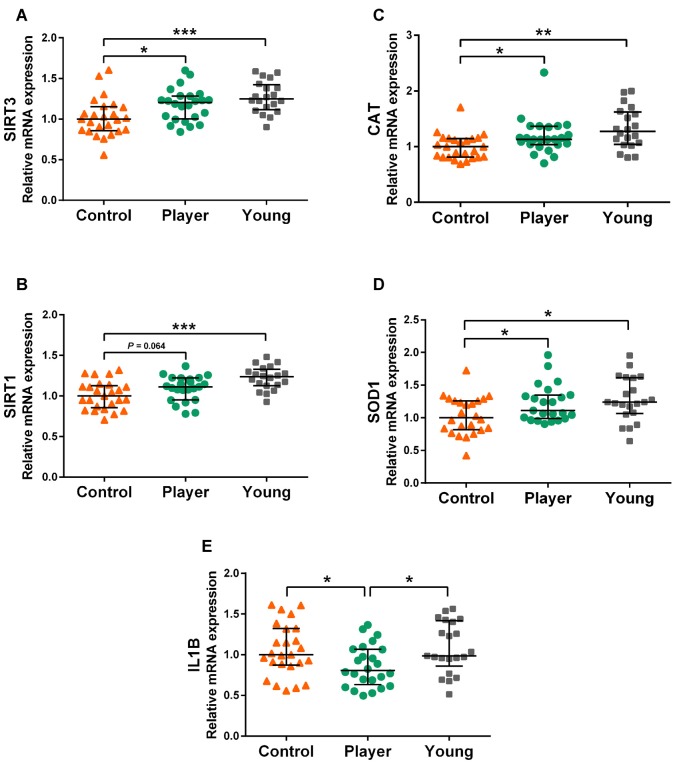
Gene expression levels of SIRT3 **(A)**, SIRT1 **(B)**, CAT **(C)**, SOD1 **(D)**, IL1B **(E)** in whole blood mRNA. Data are presented as median [interquartile range (IQR)]. Statistics: Kruskal–Wallis followed by Dunn’s test, *P < 0.05, **P < 0.01, ***P < 0.001.

Genes of the sirtuin family SIRT3 and SIRT1 and some downstream-related genes showed differential expression between middle-aged rugby players and middle-aged controls. Expression levels of genes SIRT3 and SIRT1, antioxidant genes CAT and SOD1 and pro-inflammatory gene IL1B are shown in graphs Figures 1A–E, respectively. The middle-aged control group showed significant lower expression of SIRT3, SIRT1, CAT and SOD1 than the young controls. Strikingly, the middle-aged rugby players maintained SIRT3, SIRT1, CAT and SOD1 expression at the same level as the young controls. The difference between both middle-aged groups was statistically significant for SIRT3, CAT and SOD1 and of borderline significance for SIRT1 (*P* = 0.064). Furthermore, middle-aged players showed statistically significant lower expression of IL1B than the other two groups.

**Figure 2:**
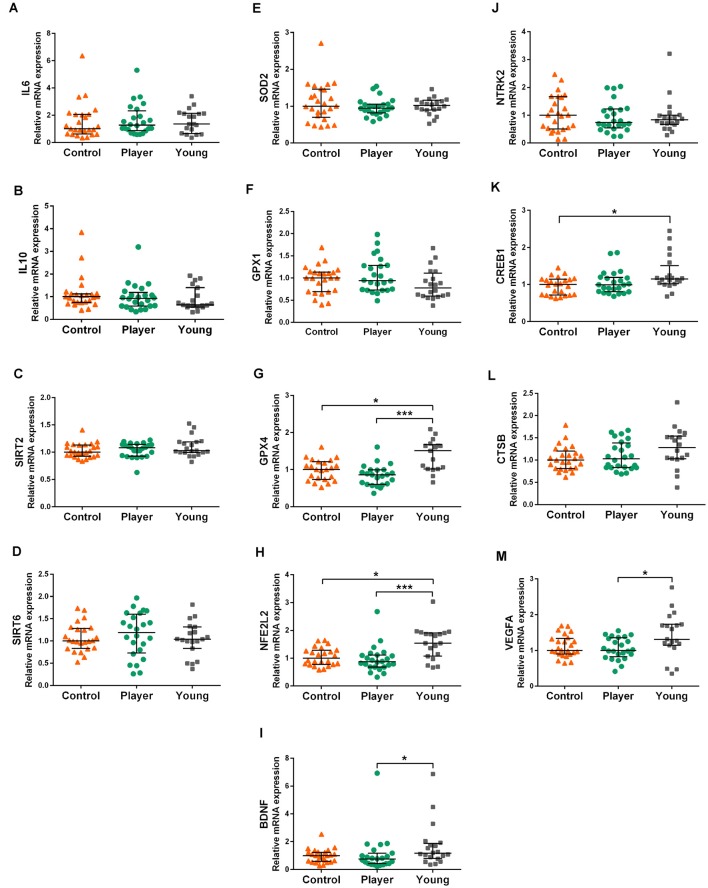
Gene expression levels of IL6 **(A)**, IL10 **(B)** SIRT2 **(C)**, SIRT6 **(D)**, SOD2 **(E)**, GPX1 **(F)**, GPX4 **(G)**, NFE2L2 **(H)** BDNF **(I)**, NTRK2 **(J)**, CREB1 **(K)**, CTSB **(L)** and VEGFA **(M)** in whole blood mRNA. Data are presented as median (IQR). Statistics: Kruskal–Wallis followed by Dunn’s test, *P < 0.05, ***P < 0.001.

The results of analyzed genes that did not show differences in expression between both middle-aged groups are shown in Figure 2. Expression of pro-inflammatory cytokine IL6, anti-inflammatory cytokine IL10, sirtuins SIRT2 and SIRT6, antioxidants SOD2 and GPX1 are displayed in graphs Figures 2A–F, respectively. No differences in expression among the three study groups were detected for these genes. Expression of the antioxidant-related genes GPX4 and NFE2L2 and tropism-related genes BDNF, NTRK2, CREB1, CTSB, and VEGFA are displayed in graphs Figures 2G–M, respectively. Both middle-aged groups showed lower expression of GPX4 and NFE2L2 than the young controls. The middle-aged control group showed lower expression in the signaling factor CREB1 than the young controls. The player group showed a lower expression in BDNF and VEGFA than the young controls. Indeed, the trophic factor genes BDNF, CTSB and VEGFA showed a tendency to decrease in both middle-age groups. No differences among groups were detected for the BDNF receptor gene NTRK2.

### Superoxide Dismutase Enzymatic Activity and Interleukin 1β Protein Levels in Plasma

Decreased SOD1 gene expression in whole blood of middle-aged control group did not induce a significant decrease in SOD enzymatic activity in the corresponding plasma samples, but only a minor trend. Results of SOD activity for Control, Player and Young groups were respectively: 66.4 (62.5,69.7), 67.7 (64.2,70.6) and 68.3 (65.8,70.5), expressed as median (IQR). The Kruskal–Wallis test was not significant.

Changes of IL1B protein levels showed borderline significant changes suggesting higher levels of this cytokine in middle-aged controls. Results for Control, Player and Young groups were respectively: 0.72 (0.13,2.64), 0.34 (0.06,0.88) and 0.07 (0.03,0.10), expressed as median (IQR).The Kruskal–Wallis test significance was *P* = 0.0548; Mann–Whitney test of paired data showed statistical differences between middle-aged controls and young controls (*P* < 0.05).

### Neuropsychological Characterization

All middle-aged subjects demonstrated normality in their responses for the categories of memory, attention, psychomotor speed, executive functions, visual processing, and premorbid intelligence. Data are shown in Table 3A for standard tests and Table 3B for the computerized CANTAB battery. The rugby players showed significantly higher scores than the controls in the immediate memory recall of the FCSRT as previously reported (De la Rosa et al., 2019). No statistical differences were detected in any of the other parameters tested. However, the player group showed a trend (*P* = 0.078) of better executive function than the controls in the TMT Part B test. Neuropsychological results of both groups, controls and rugby players, were used here for statistical correlation study.

**Table 3A:** Neuropsychological testing.

	Middle-aged controls	Middle-aged rugby players	*P*
	*N* = 25	*N* = 24	
**Declarative memory**			
FCSRT, Immediate free recall^1^	18.2 ± 5.1	21.8 ± 5.9*	0.0270
FCSRT, Immediate cued recall^1^	27.3 ± 5.1	31.1 ± 5.2*	0.0123
FCSRT, Delayed free recall	8.3 ± 2.2	9.3 ± 2.8	0.1662
FCSRT, Delayed cued recall	12.1 ± 2.5	13.2 ± 2.3	0.1318
**Attention and psychomotor speed**			
TMT, Part A	27.2 ± 7.5	25.8 ± 6.1	0.4646
SDMT	48.4 ± 8.8	51. 7 ± 8.7	0.2023
WAIS IV, Direct digits	9.2 ± 1.9	10.0 ± 2.4	0.2194
**Executive functions**			
TMT, Part B	68.0 ± 19.1	58.4 ± 18.0	0.0781
WAIS IV, Inverse digits	6.4 ± 2.7	6. 7 ± 2.4	0.6745
Verbal fluency, Semantic	20.8 ± 4.7	21.4 ± 5.2	0.6635
Verbal fluency, Phonemic	43.8 ± 12.8	46.3 ± 11.3	0.4682
Stroop test	51.8 ± 7.1	52.8 ± 5.7	0.5777
**Premorbid intelligence level**			
WAIS IV, Vocabulary	14.1 ± 1.8	13.6 ± 2.1	0.4219

Data are presented as mean ± SD. Statistics: Two-tailed *t*-test, *P < 0.05 compared to Middle-aged control group. Abbreviations: FCSRT, Free and Cued Selective Reminding Test; TMT, Trail Making Test; SDMT, Symbol Digit Modality Test; WAIS-IV, Wechsler Adult Intelligence Scale fourth edition; ^1^results for FCSRT immediate recall were reported in De la Rosa et al. (2019).

**Table 3B:** Cambridge Neuropsychological Test Automated Battery (CANTAB).

	Middle-aged controls	Middle-aged rugby players	*P*
	*N* = 25	*N* = 24	
**Spatial working memory (SWM)**			
Latency to first response	2,229 ± 1,245	2,215 ± 1,273	0.9620
Strategy	41.04 ± 6.28	42.00 ± 7.22	0.6185
Double Errors	0.08 ± 0.28	0.04 ± 0.21	0.9990
**Paired association learning (PAL)**	
First trial memory score	11.56 ± 3.70	12.65 ± 3.13	0.3001
Trials to success	2.34 ± 1.03	2.26 ± 0.92	0.8732
Stages completed	3.68 ± 0.56	3.78 ± 0.42	0.6467
**Rapid visual information processing (RPV)**	
Total hits	37.44 ± 7.95	37.30 ± 8.99	0.9877
Latency	449.2 ± 82.59	442.6 ± 62.15	0.8642
Correct Rejections	506.5 ± 20.14	507.7 ± 18.96	0.9959

*Data are presented as median (IQR). Statistics: Mann–Whitney test*.

### Statistical Correlations Between Categories of Parameters

**Figure 3:**
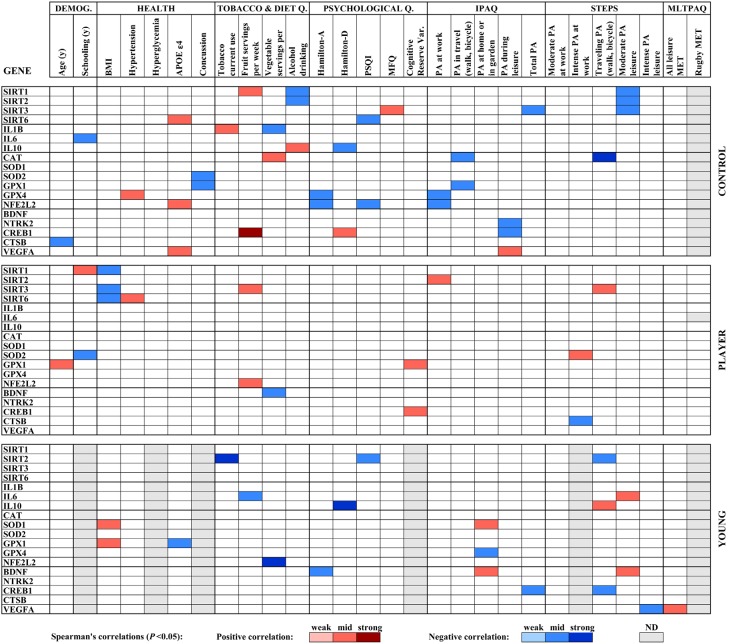
Heat map built with Spearman’s correlations between gene expression analysis and sociodemographic, health, diet and physical activity parameters obtained by standardized questionnaires. Potency of significant correlations (*P* < 0.05) were defined as weak (/R/ < 0.400), moderate (0.400 ≤ /R/ < 0.600) or strong (/R/ ≥ 0.600), as shown by color code.

The results of the analysis of correlations of gene expression with age, education, health and lifestyle characteristics for the three groups of the study; middle-aged controls, middle-aged rugby players and young controls, were used to build a heat map displayed in Figure 3. Significant Spearman’s correlations showed values between /R/ = 0.41, *P* < 0.05 and /R/ = 0.74, *P* < 0.001. To highlight some of the findings, Spearman’s analysis of sirtuin genes with diverse physical activity parameters showed a scattered positive correlation with SIRT2 and SIRT3 in players, negative correlations for SIRT1, SIRT2 and SIRT3 in middle-aged controls and negative correlations for SIRT2 in the young controls. In both control groups, IL10 levels positively correlated with fewer depression symptoms according to Hamilton-D. IL1B positively correlated with tobacco use and negatively correlated with vegetal servings in middle-aged controls. IL6 negatively correlated with the fruit servings in the diet of young controls. Antioxidant genes showed scattered negative correlations with physical activity parameters for CAT, GPX1, and GPX4 in middle-aged control group and positive correlations for SOD1 with home physical activity in young controls and for SOD2 with intense leisure physical activity in player group. BDNF was positively correlated with physical activity in the young control group. Other scattered correlations with physical activity included negative ones for CTSB in Players, positive ones for VEGFA in middle-aged controls and irregular VEGFA correlations in young controls. CREB1 negatively correlated with some parameters of physical activity in both control groups.

**Figure 4:**
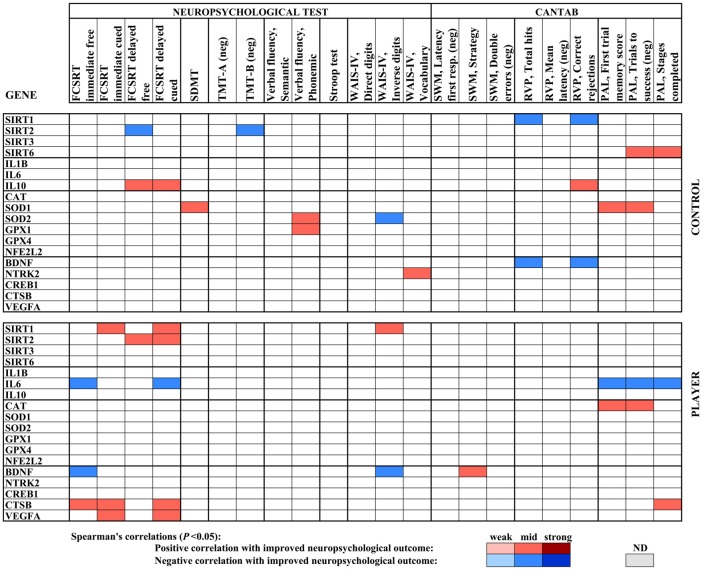
Heat map built with Spearman’s correlations between gene expression analysis and neuropsychological parameters of the middle age cohort. The sign of the correlation coefficient is reversed where the better neuropsychological outcome is measured by the lower value of the parameter, as indicated (neg). Potency of significant correlations (*P* < 0.05) were defined as weak (/R/ < 0.400), moderate (0.400 ≤ /R/ < 0.600) or strong (/R/ ≥ 0.600), as shown by color code.

A heat map compiled with the significant correlations in gene expression with neuropsychological testing for both middle-aged groups, controls, and rugby players, as shown in Figure 4. Significant Spearman’s correlations showed values ranged from /R/ = 0.41, *P* < 0.05, to /R/ = 0.58, *P* < 0.01. The player group showed positive correlations of SIRT1 for both cued subtests of FCSRT and WAIS-IV Inverse digits, as well as positive correlations for SIRT2 with both subtests of delayed FCSRT. However, the middle-aged control group showed scattered negative correlations for both genes and positive correlations between SIRT6 and PAL parameters. In the inflammatory status, IL6 expression negatively correlated with FCSRT and CANTAB PAL test parameters in the player group, whereas IL10 correlated with both cued subtests of FCSRT in the controls. Antioxidant enzyme genes in both groups also correlated positively with improved neuropsychological outcomes, such as the CAT gene with PAL parameters in players, SOD1 with SDMT and PAL parameters in controls, and SOD2 and GPX1 with verbal fluency also in controls. BDNF showed some generally negative correlations with neuropsychological scores in both groups, whereas CSTB and VEGFA showed a positive correlation with FCRT parameters in the player group.

**Figure 5:**
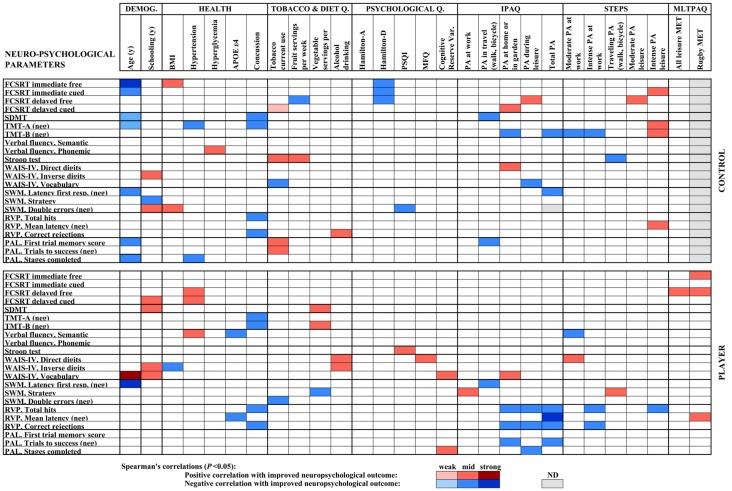
Heat map built with Spearman’s correlations between neuropsychological parameters of the middle-age cohort and sociodemographic, healthy diet and physical activity parameters obtained by standardized questionnaires. The sign of the correlation coefficient is reversed where the better neuropsychological outcome is measured by the lower value of the parameter, as indicated (neg). Potency of significant correlations (*P* < 0.05) was defined as weak (/R/ < 0.400), moderate (0.400 ≤ /R/ < 0.600) or strong (/R/ ≥ 0.600), as shown by color code.

The heat map built with the correlations between neuropsychological parameters and age, education, health and lifestyle characteristics for middle-age controls and rugby players is shown in Figure 5. Significant correlations showed values ranging from /R/ = 0.41, *P* < 0.05, to /R/ = 0.70, *P* < 0.01. Several parameters of higher physical activity positively correlated with a higher memory level in the FCSRT, which may underlie the significant improvement shown in the rugby player group. In the player group, yearly MET-h of rugby activity (MLTPAQ) correlated positively with both immediate and delayed free recall of FCSRT. MET-h of total leisure activity also correlated positively with the later measure. In the control group, STEPS and IPAQ parameters of leisure and domestic activity correlated positively with either cued or free delayed recalls. Correlation of physical activity parameters with other neuropsychological tests showed irregular outcomes. Namely, the control group showed a positive correlation between STEPS intense leisure physical activity and TMT-A performance. In the WAIS-IV, the Direct digits subtest correlated positively with IPAQ domestic physical activity in the controls, and with STEPS moderate physical activity at work in the player group. The WAIS-IV Vocabulary subtest positively correlated with IPAQ domestic physical activity in the player group. In the CANTAB battery, the SWM strategy showed positive correlations with IPAQ work and STEPS transportation in the player group and RPV latency decreased with MET-h of rugby activity in MLTPAQ. Furthermore, decreased RPV latency correlated with STEPS intense leisure activity in the controls. Among some scattered negative correlations, several IPAQ and STEPS physical activity parameters correlated with a poorer outcome in the RVP subtest of CANTAB in the player group. The health records for the player group showed a higher number of subjects with one or more episodes of a concussion than those in the control group. Concussions correlated negatively with performance in SDMT and TMT-A in the controls, TMT-A and B in the players, and RPV in both groups. Parameters of BMI, hypertension, and hyperglycemia showed irregular trends in the correlation with the neuropsychological test outcome. Presence of one APOE ε4 allele only correlated with lower semantic fluency and higher RPV latency in the player group. Higher depression scores in the Hamilton-D scale correlated negatively with FCSRT memory scores in three subtests in the control group. Poor sleeping habits according to the PSQI scale correlated with more CANTAB SWM errors in the controls. In diet habits, tobacco use showed an irregular outcome. Frequency of alcoholic drink consumption positively correlated with the score of Digit subtests of WAIS-IV in players and RPV correct rejections in controls. Increased vegetal servings correlated positively with better response in WAIS-IV Inverse digits but the worst strategy in SWM. As expected, increased years of schooling generally showed a positive correlation with better neuropsychological results; Specifically, with higher scores in the delayed cued memory subtest of FCSRT, SDMT and Vocabulary subtest of WAIS-IV in Players, with higher scores of WAIS-IV Inverse digits in both controls and players and with lower errors in the SWM subtest of CANTAB in controls. Lastly, age within the group negatively correlated with the neuropsychological performance in a number of parameters in middle-age controls but showed lower effect in rugby players.

## Discussion

Middle-aged amateur rugby players that had a long-term record of intense physical activity at leisure time, showed a distinct profile in whole blood gene expression compared to the age-matched controls with low sports activity. Furthermore, differences in the physical activity parameters showed some correlations with gene expression that supported the differential expression profile between rugby players and age-matched controls.

The central expression changes in the panel of 18 selected genes analyzed are those involving sirtuin genes SIRT1 and SIRT3. Rugby players maintained young-like levels of the sirtuin gene expression, whereas low-activity middle-aged controls showed a significant decrease. These findings in healthy middle-aged men may support the relevance of SIRT1 and SIRT3 as biomarkers of resilience against cognitive decline and/or in age-related frailty. Correlations of the expression of these genes with diverse physical activity parameters and neuropsychological parameters were generally positive in the player group and negative in the control subjects. The decreased expression of SIRT1 and SIRT3 genes in middle-aged controls compared to young controls is in agreement with previous reports on decreased serum levels of SIRT1 and SIRT3 proteins with aging (Kumar et al., 2014). These authors analyzed male subjects aged 60–80 years and proposed the lower circulating levels of SIRT1 and SIRT3 as markers of frailty. Much lower SIRT1 blood levels have been reported in cognitively impaired elders and AD patients (Kumar et al., 2013). In AD brains, there is a decrease in both the protein and mRNA of SIRT1 (Julien et al., 2009) and SIRT3 (Lee et al., 2018). Furthermore, decreases in SIRT3 have been associated with age-related hearing loss (Zeng et al., 2014) and age-exacerbated heart damage (Porter et al., 2014). The group of rugby players maintained SIRT1 and SIRT3 expression at the level of the young subjects. The significantly increased physical activity of the player group compared to that of the age-matched controls may be the cause of their rejuvenation-like gene expression in blood cells. Similarly, male and female master athletes aged 60–70 years have shown higher mRNA and protein levels of SIRT1 and SIRT3 in skeletal muscle than aged-matched controls (Koltai et al., 2018). It is plausible that the levels of sirtuins in veteran sport practitioners, both track and field athletes, and rugby players, were also higher in the brain. Accordingly, experimental studies have demonstrated that chronic running exercise increases the protein levels of SIRT1 (Revilla et al., 2014) and SIRT3 (Bo et al., 2014) in the hippocampus of AD transgenic mice up to the levels of wild-type mice in conjunction with learning and memory normalization. The molecular mechanisms underlying the neuroprotective functions of SIRT1 have been studied in animal models. This nuclear-located sirtuin senses nutrient or stress signaling and responds activating pathways of synaptic plasticity and cognition (Gao et al., 2010; Michán et al., 2010). SIRT1 overexpression in the hippocampus of either AD transgenic mice or wild type mice induces neurotrophic factors, improves proteostasis of abnormal proteins and induces cognitive enhancement (Corpas et al., 2017). The later results that show improvement of neuroprotective mechanisms in normal healthy mice indicate the potential for brain resilience of SIRT1. Indeed, here we found that SIRT1 gene expression in whole blood positively correlated with parameters of memory and executive functions in players, but not in middle-aged controls. Furthermore, SIRT1 activates anti-inflammatory and antioxidant pathways, improves insulin sensitivity and other metabolic aspects, and delays senescence in brain and peripheral organs (Haigis and Sinclair, 2010). SIRT3 controls many functional aspects of mitochondria, the energy generator of the cell, and therefore has a significant role in healthy aging (McDonnell et al., 2015). SIRT3 is involved in cell metabolism and DNA repair in a variety of tissues, and also activates antioxidant genes such as SOD2 and CAT (Ansari et al., 2017). Its neuroprotective mechanisms have been less studied than those of SIRT1. However, SIRT3 has shown to mediate adaptive response of neurons to metabolic and oxidative stress (Cheng et al., 2016; Li et al., 2018). Therefore, similarly to SIRT1, SIRT3 activation would increase resilience against neurodegeneration. Not unexpectedly, there is a close relationship between both SIRT1 and SIRT3. SIRT3 is a substrate for SIRT1 deacetylation and therefore its activation level may be directly modulated by SIRT1 (Kwon et al., 2017). In addition, they deacetylate some homologous substrates present in their corresponding cell compartments, nucleus and mitochondria (Hirschey et al., 2011), and may act cooperatively in response to inducing factors (Bell and Guarente, 2011) or against pathological conditions (Kwon et al., 2017; Chen et al., 2018). Therefore, we propose that the maintenance of the SIRT1-SIRT3 axis functionality, both in the brain and peripheral tissues, is a molecular mechanism that contributes to the acquisition of resilience through long-term physical activity. SIRT2 and SIRT6 did not show differential expression based on physical activity or age of the groups. The role of SIRT2 in the regulation of aging is not clarified. SIRT2 protein levels decrease in blood serum of frail patients, but less significantly than SIRT1 and SIRT3 (Kumar et al., 2014), and it was suggested as being a marker of senescence and neurodegeneration (Theendakara et al., 2013). However, it also may promote longevity (North et al., 2014). SIRT6 is an anti-aging sirtuin that regulates metabolism and genome stability (Kuang et al., 2018). Its functions of DNA repair and cell survival are particularly neuroprotective in the brain, where it decreases with aging and AD (Kaluski et al., 2017). Absence of any change in the expression of SIRT2 and SIRT6 gene may, therefore, be indicative of the health status of all the subjects analyzed.

Antioxidant genes CAT and SOD1 showed a striking similarity to SIRT1 and SIRT3 in their response to physical activity. Players showed a young-like level of CAT and SOD1 expression and middle-aged controls a significant decrease of them. They might be activated downstream of sirtuin genes in response to physical exercise. Activities of CAT and SOD enzymes are known to increase in rodent brain submitted to physical training (de Souza et al., 2019). Catalase is a first-line antioxidant that decomposes hydrogen peroxide. It may have an anti-aging role as seen by increased CAT gene expression in the liver of long-lived mouse models and decreased CAT expression in short-lived models (Brown-Borg and Rakoczy, 2000). Here we demonstrated a decrease in gene expression in whole blood with age. Increased CAT expression might be neuroprotective given that, for instance, CAT expression correlated positively with visual memory in the player group. The cytosolic enzyme Cu/ZnSOD is the main responsible for the decomposition of superoxide radicals. It has been reported a decrease of Cu/ZnSOD levels in the brain of aged AD transgenic female mice that were normalized by a neuroprotective therapy of physical exercise (García-Mesa et al., 2016). We demonstrated gene expression changes in blood cells but we did not detect changes of SOD enzymatic activity, indicating that oxidative stress processes at middle age are mild. Indeed, our previous study showed no differences in plasma oxidized proteins or lipids between middle-aged controls and young controls (De la Rosa et al., 2019). The genes SOD2 and GPX1 that code for the antioxidant enzymes MnSOD and GPx1 did not show differences in expression levels with age or physical activity in whole blood. MnSOD decomposes superoxide anion in the mitochondria. GPx1 is the more abundant GPx isoenzyme and reduces mainly hydroperoxides in the cytoplasm. Both enzymes have shown altered brain activity levels in animal models of AD with elevated oxidative stress (García-Mesa et al., 2016). Therefore, lack of changes in these widely expressed antioxidant genes would also support the absence of oxidative processes of neurodegeneration or other chronic diseases in apparently healthy middle-aged subjects. However, we found a decrease in the expression of the gene GPX4 coding for the GPx4 isoenzyme in both middle-aged controls and players. Enzymatic levels of this isoform are much lower than those of GPx1, although it shows high affinity for lipid peroxides and has been suggested as being neuroprotective (Cardoso et al., 2017). Further studies are needed to understand the relevance of GPX4 expression changes in aging in diverse tissues. The expression profile of NFE2L2 was also lower in both middle-aged groups. There is no consensus on the age-related changes of the protein levels of Nrf2 and its signaling activity but the decline of this pathway induces premature aging (Kubben et al., 2016).

The cytokine IL1B is an important mediator of the inflammatory response. Its decreased level in the middle-aged rugby players as compared to young controls confirmed the reported anti-inflammatory effect of physical activity at older ages (Jankord and Jemiolo, 2004). Although no differences were found between middle-aged controls and young controls in the gene expression, protein levels of IL1B in plasma showed a trend to increase in middle-aged controls as compared to young control. Therefore, the changes of age-related inflammation are probably not yet clearly detectable at middle age. No differences were found in the levels of gene expression of IL6 and IL10 among the groups. Gene expression of pro-inflammatory or anti-inflammatory markers may be lower in the blood than in other compartments (Pilling et al., 2015). However, it is interesting to note that the expression level of pro-inflammatory cytokine IL6 negatively correlated with memory and visual processing parameters in the rugby players and that the level of expression of anti-inflammatory cytokine IL10 positively correlated with scores for memory and attention and executive function in controls. Anti-inflammatory changes may be downstream of sirtuin activation through NF-κB modulation.

Expression levels of analyzed neurotrophic factors and related genes in whole blood did not increase in the middle-aged rugby players as compared to middle-aged control that would support a further contribution to the proposed anti-aging effects of physical exercise. The expression of the trophic factor genes BDNF and VEGFA and the myokine gene CSTB showed a tendency to decrease at middle age compared to young age. Such behavior was significant in the players’ group for BDNF and VEGFA. No changes were detected for the BDNF receptor gene TRK. The expression of CREB1 also tended to decrease with age, with such a change being most significant for the control group. CREB1 signaling is activated after endurance exercise to induced downstream protective genes (Neubauer et al., 2014). CREB1 is a transcriptional activator involved in sirtuin signaling as well as in the regulation of neurotrophic factors such as BDNF. BDNF is synthesized in the brain but also in muscle and other peripheral compartments. BDNF has been suggested as being a key mediator for increases in the volume of the hippocampus and related cognitive benefits of physical exercise (Erickson et al., 2011). The role of the cysteine protease CSTB in the brain is poorly understood, but its release from skeletal muscle following exercise could induce cognitive benefits through several pathways including induction of BDNF and neurogenesis (Moon et al., 2016). VEGFA is released mainly from the endothelial cells of muscle capillaries following acute exercise and it acts synergistically with the cascade of trophic factors (Archer, 2011). These analyses were performed under resting conditions, where we had previously reported a decrease in serum levels of BDNF and CTSB proteins in young and middle-aged trained men as compared to age-matched sedentary subjects (De la Rosa et al., 2019). Therefore, we cannot discard that the transduction of some trophic factors sensitive to the exercise may decrease during the resting period as found here for BDNF and VEGF. However, VEGFA and CTSB expression correlated positively with declarative memory parameters in the player group, although not BDNF, TRK or CREB1. We could not determine the gene expression of another two trophic factors induced by physical activity; IGF1 which is deeply involved in the neurogenesis mediated by exercise (Trejo et al., 2001) and the neuroprotective GDNF (Revilla et al., 2014), because they had low expression in the whole blood samples.

The absence of differences in sociodemographic variables, and health and lifestyle parameters other than leisure physical activity between both middle-aged groups indicates that long-term engagement in playing amateur rugby may underlie the differential gene expression. As discussed above, the changes found in the sirtuin genes in blood cells are in agreement with the results in brain tissue after physical exercise reported in previous studies in experimental models of aging and neurodegeneration. Furthermore, sirtuins have experimentally proved pro-cognitive effects. Here, the expression levels of genes SIRT1, SIRT3, and downstream CAT and SOD1 in the player group were similar to those in the young controls, but middle-aged controls showed a significant decrease compared to young age levels. These results paralleled a better memory outcome in rugby players that in middle-aged controls. We speculate that the maintenance of the peripheral axis SIRT1-SIRT3 in middle-aged active men reflects the brain status and it may contribute to the prevention of age-associated cognitive loss and neurodegeneration through physical exercise.

It is likely that the findings in this study can be extended to middle-aged women. We have not been able to enroll rugby player women, but previous studies have reported benefices of physical activity in the cognitive health of aged women (Yaffe et al., 2001; Lin et al., 2019). Furthermore, as discussed above, old female master athletes have shown higher mRNA and protein levels of SIRT1 and SIRT3 in skeletal muscle than aged-matched controls (Koltai et al., 2018) suggesting activation of SIRT1-SIRT3 axis. Therefore, no differential gender response is anticipated.

Not less important, a leisure sport activity practiced in a team such as amateur rugby might facilitate to comply with the volume of physical activity required to maintain health and wellbeing. In most medium and high-income countries, there is an urgent call to implement programs, practices, and policies of physical exercise at all ages (Piercy et al., 2018). Resilience findings at middle age in amateur rugby players can help clinicians monitor and improve the cognitive health of the aging population.

## Limitations of the Study

Incidences of concussion in the player group were higher than in the controls and we cannot exclude some effect of this factor in the parameters analyzed. Nevertheless, negative correlations between some IPAQ and STEPS parameters and CANTAB RPV and PAL performance in the player group may suggest the presence of specific minor impairments in the subjects affected by one or more episodes of mild brain trauma. However, these mild accidents did not cause worsening of the neuropsychological response, as in contrast, rugby players showed some memory improvements compared to age-matched controls. Although rugby is a contact sport, the risk of moderate or severe traumatic brain injury is low. Rugby playing is generally less harsh than American football and no effects of the later have been reported for all-cause mortality in retired football players (Venkataramani et al., 2018).

The volume of physical exercise of each participant in the study was obtained from standardized questionnaires. In the absence of physiological measures of fitness, we cannot discard some inaccuracies in the time or the intensity of the exercise performed.

The cross-sectional design of the study introduces some weakness because it may preclude the detection of possible bias in the groups. However, the long-term sports activity clearly differentiates the rugby player group from the control group. Middle-aged controls had lower leisure physical activity but otherwise had similar behaviors and educational level. Furthermore, in the players’ group, whole year METs-h spent in rugby playing correlated with higher declarative memory in FCSRT; thus, supporting the positive effect of the sport on this collective of long-time practitioners of amateur rugby.

## Conclusions

This study supports the beneficial effect of long-term practice of leisure sport as an anti-aging and neuroprotective lifestyle and provides further evidence of SIRT1 and SIRT3 gene activation by physical activity.

The young-like levels of SIRT1, SIRT3, CAT, and SOD1 gene expression and the lower expression of IL1B, determined in peripheral blood of middle-aged amateur rugby players, suggest that the SIRT1-SIRT3 axis and downstream genes may contribute to the beneficial effects of physical activity. Similar changes in the brain may be a factor of anti-age resilience.

## Data Availability Statement

The raw data supporting the conclusions of this article will be made available by the authors, without undue reservation, to any qualified researcher.

## Ethics Statement

The study was approved by the Ethics Committee of the Hospital Clinic de Barcelona, Spain (Reg. HCB/2014/0759). Written informed consent was obtained from all participants and all procedures were conducted in accordance with the 1964 Declaration of Helsinki and its later amendments.

## Author Contributions

JV, ER-F, MP, DB-F, MG-C, and CS contributed to the conception and design of the study. DB-F, MG-C, and CS obtained ethical approval and jointly supervised the study. ES conducted the lifestyle questioning and neuropsychological testing. RC, AR, and MO obtained and processed blood samples. RC, AR, SS, CG-F, EC, and CS did the experimental analysis and data processing. CS wrote the manuscript draft. All authors contributed to manuscript revision, read and approved the submitted version.

## Conflict of Interest

The authors declare that the research was conducted in the absence of any commercial or financial relationships that could be construed as a potential conflict of interest.
