# Supplementary material for: Peripheral Maintenance of the Axis SIRT1-SIRT3 at Youth Level May Contribute to Brain Resilience in Middle-Aged Amateur Rugby Players
Source: Front Aging Neurosci. 2019 Dec 17;11:352. doi: 10.3389/fnagi.2019.00352 (PMC6951402; doi:10.3389/fnagi.2019.00352)
Supplement: Supplementary file 1 [file Table_1.DOCX]

**Supplementary Table 1** TaqMan FAM-labeled probes for mRNA analysis

| **Gene Symbol** | **Description** | **TaqMan Assay ID** | **NCBI RefSeq** |
| --- | --- | --- | --- |
| B2M | beta-2-Microglobulin | Hs00984230_m1 | NM_004048.3 |
| BDNF | Brain derived neurotrophic factor | Hs02718934_s1 | NM_001143805.1 |
| CAT | Catalase | Hs00156308_m1 | NM_001752.3 |
| CREB1 | cAMP responsive element binding protein 1 | Hs00231713_m1 | NM_001320793.1 |
| CTSB | Cathepsin B | Hs00947433_m1 | NM_001317237.1 |
| GPX1 | Glutathione peroxidase 1 | Hs00829989_gH | -- |
| GPX4 | Glutathione peroxidase 4 | Hs00989766_g1 | NM_001039847.2 |
| IL1B | Interleukin 1 beta | Hs01555410_m1 | NM_000576.3 |
| IL6 | Interleukin 6 | Hs00174131_m1 | NM_000600.4 |
| IL10 | Interleukin 10 | Hs00961622_m1 | NM_000572.2 |
| NFE2L2 | Nuclear factor, erythroid 2 like 2 | Hs00975961_g1 | NM_001145412.3 |
| NTRK2 | Neurotrophic receptor tyrosine kinase 2 | Hs00178811_m1 | NM_001007097.2 |
| PGK1 | Phosphoglycerate kinase 1 | Hs00943178_g1 | NM_000291.4 |
| SIRT1 | Sirtuin 1 | Hs01009006_m1 | NM_001142498.1 |
| SIRT2 | Sirtuin 2 | Hs00247263_m1 | NM_001193286.1 |
| SIRT3 | Sirtuin 3 | Hs00953477_m1 | NM_001017524.2 |
| SIRT6 | Sirtuin 6 | Hs00966002_m1 | NM_001193285.2 |
| SOD1 | Superoxide dismutase 1 | Hs00533490_m1 | NM_000454.4 |
| SOD2 | Superoxide dismutase 2, mitochondrial | Hs00167309_m1 | NM_000636.3 |
| VEGFA | Vascular endothelial growth factor A | Hs00900055_m1 | NM_001025366.2 |
